# Comparing product quality between translation and paraphrasing: Using NLP-assisted evaluation frameworks

**DOI:** 10.3389/fpsyg.2022.1048132

**Published:** 2022-11-25

**Authors:** Tianyi Han, Dechao Li, Xingcheng Ma, Nan Hu

**Affiliations:** ^1^Department of Chinese and Bilingual Studies, The Hong Kong Polytechnic University, Kowloon, Hong Kong SAR, China; ^2^School of Foreign Languages, Southeast University, Nanjing, China; ^3^Institute of Chinese Information Processing, Beijing Normal University, Beijing, China

**Keywords:** translation quality assessment, paraphrasing quality assessment, natural language processing, product quality, linguistic features

## Abstract

Translation and paraphrasing, as typical forms in second language (L2) communication, have been considered effective learning methods in second language acquisition (SLA). While many studies have investigated their similarities and differences in a process-oriented approach, little attention has been paid to the correlation in product quality between them, probably due to difficulties in assessing the quality of translation and paraphrasing. Current quality evaluation methods tend to be either subjective and one-sided or lack consistency and standardization. To address these limitations, we proposed preliminary evaluation frameworks for translation and paraphrasing by incorporating indices from natural language processing (NLP) tools into teachers’ rating rubrics and further compared the product quality of the two activities. Twenty-nine translators were recruited to perform a translation task (translating from Chinese to English) and a paraphrasing task (paraphrasing in English). Their output products were recorded by key-logging technique and graded by three professional translation teachers by using a 10-point Likert Scale. This rating process adopted rubrics consisting of both holistic and analytical assessments. Besides, indices containing textual features from lexical and syntactic levels were extracted from TAASSC and TAALES. We identified indices that effectively predicted product quality using Pearson’s correlation analysis and combined them with expert evaluation rubrics to establish NLP-assisted evaluation frameworks for translation and paraphrasing. With the help of these frameworks, we found a closely related performance between the two tasks, evidenced by several shared predictive indices in lexical sophistication and strong positive correlations between translated and paraphrased text quality according to all the rating metrics. These similarities suggest a shared language competence and mental strategies in different types of translation activities and perhaps in other forms of language tasks. Meanwhile, we also observed differences in the most salient textual features between translations and paraphrases, mainly due to the different processing costs required by the two tasks. These findings enrich our understanding of the shared ground and divergences in product quality between translation and paraphrasing and shed light on the pedagogical application of translation activities in classroom teaching. Moreover, the proposed evaluation framework can also bring insights into the development of standardized evaluation frameworks in translation and paraphrasing in the future.

## Introduction

In recent years, research in L2 learning has shown a growing positive attitude toward the role of translation activities in language teaching (Cappelli, 2016). As defined by Jakobson (1959), interlingual translation and intra-lingual paraphrasing are two typical types of translation activities. A number of studies have been conducted to explore the overlaps and divergences between these two activities (Whyatt et al., 2016, 2017; Whyatt and Naranowicz, 2020; Ma et al., 2022). However, most of the attention has been paid to the comparison from a cognitive perspective. Little is known about whether the performances of the two tasks are highly correlated or radically different, as is shown by the product quality of these two activities.

One premise for investigating product quality is to find a suitable and comprehensive framework for quality evaluation. However, there seems to be no consensus on a specific criterion for translation and paraphrasing tasks with a communicative purpose, and current methods for translation and paraphrasing quality assessment have specific problems (Han, 2020; Shalamov et al., 2021). In translation quality assessment (TQA), the widely adopted methods of error analysis and rubric scoring tend to be subjective and unreliable. By contrast, current methods in paraphrasing quality assessment (PQA) are mostly automatic evaluation models, which assess the paraphrasing quality solely based on linguistic and semantic similarity regardless of the communicative function of the product. These limitations call for developments in the evaluation criteria in TQA and PQA.

In light of the research gap in the comparison between translation and paraphrasing, as well as the limitations on quality evaluation methods, the current study proposes preliminary evaluation frameworks for TQA and PQA by incorporating indices of textual features into translation teacher’s evaluation rubrics and further investigates the correlation in product quality between translation and paraphrasing. Our contributions are expected to deepen the understanding of the resemblances and divergences between translation and paraphrasing and hopefully provide practical insights into translation and paraphrasing quality assessment and classroom teaching.

### Translation activities in the development of L2 competence

Contemporary trends in SLA have led to a proliferation of studies that advocate integrating traditional grammar instruction into real communicative situations (Brown, 1994; Colina, 2002; Machida, 2011). One primary goal of this communicative approach in language teaching is to improve students’ ability of appropriate meaning negotiation in a situated context. In line with this trend, translation activities, as typical ways of communicative language use, are proposed as ‘unique forms of second language education’ (Kiraly, 1995; Colina, 2002). According to Jakobson (1959), translation activities encompass three types: interlingual, intra-lingual, and intersemiotic. Among them, interlingual translation refers to a reformulation of meaning across cultural and linguistic barriers, whereas intra-lingual translation can be generally understood as using different forms of the same language to deliver a message, such as paraphrasing (Zethsen, 2009; Whyatt et al., 2017). Both activities are usually performed with the purpose of achieving a particular communicative function regarding the purpose of the task or the type of target audience (Campbell, 1998; Colina, 2002).

In the last two decades, scholars have started to propose involving translation activities in communicatively oriented foreign language classrooms (Colina, 2002; Cook, 2010; Liashuk, 2018). For intra-lingual paraphrasing (paraphrasing), a considerable amount of literature has introduced its positive role in enhancing students’ L2 reading, speaking, and writing skills (Takatsuka, 1999; Fisk and Hurst, 2003; Choy and Lee, 2012; Ramsden, 2021). Some scholars propose that paraphrasing, in a broader term, can also be perceived as a vital strategy to facilitate language comprehension and production in both classrooms and daily conversations (Takatsuka, 1999; Chen et al., 2015; Li, 2015). Similarly, interlingual translation (translation) is gradually gaining recognition as a pedagogical method in the field of language teaching, notwithstanding the strong criticism by opponents of the grammar-translation approach in SLA (Machida, 2011; Laviosa, 2014; Liao, 2016; Navidinia et al., 2019). Recent research has revealed the possibility of using translation as a pedagogical tool to benefit vocabulary building, wmorphosyntactic accuracy improvement, and intercultural awareness (Liashuk, 2018). Moreover, introducing translation strategies directly into L2 learning also helps to improve students’ reading comprehension in L2 (Davaribina and Asl, 2017). As concluded by Popescu (2013), the application of translation activities into L2 learning roots in the inherent overlap in language competence, which allows for the interrelated improvement in linguistic knowledge, cognitive competence like planning, monitoring, and execution in reading and writing, as well as communicative abilities such as stylistic fluency, intercultural awareness (Cappelli, 2016; Navidinia et al., 2019).

### The relationship between translation and paraphrasing

Defined as two types of translation activities mentioned earlier, translation and paraphrasing share some family resemblances and contrast in certain aspects (Zethsen, 2009; Whyatt et al., 2016). In general, both tasks involve a successful comprehension of an original text and a reconstruction of a target text in different linguistic forms according to specific communicative purposes. During this process, the required higher-order metacognitive skills tend to be similar and even transferrable between two tasks, such as planning, monitoring, decision-making, and self-correcting (Whyatt et al., 2017; Whyatt and Naranowicz, 2020). In addition, the reconstruction process of both tasks can be impeded by comparable linguistic and cognitive constraints such as inadequate linguistic knowledge and the restricted capacity in working memory (Kruger, 2012; Whyatt et al., 2016). These constraints lead to shared strategies employed during production, including addition, reduction, restructuring, and stylistic simplification (Zethsen, 2009; Kajzer-Wietrzny et al., 2016; Whyatt et al., 2017). Still, one notable difference between translation and paraphrasing lies in the number of activated languages, with the former switching between two languages whereas the latter only involving monolingual processing (Whyatt et al., 2016). Some studies further argue that compared to paraphrasing, translation across language barriers is inclined to induce heavier cognitive load, mainly attributed to the additional processing cost caused by cognitive control of two activated languages (Whyatt et al., 2016, 2017).

Several empirical studies have explored the similarities and differences between translation and paraphrasing (Whyatt et al., 2016, 2017; Kajzer-Wietrzny, 2019; Whyatt and Naranowicz, 2020; Ma et al., 2022). A seminal study in this area is the work of Whyatt’s team in 2016, which compares the cognitive rhythm and processing effort between translation from English to Polish and paraphrasing in polish using eye-tracking and key-logging (Whyatt et al., 2016). The authors observed an overall increased cognitive load in translation evidenced by longer fixation duration in the source text and lower processing speed in support of the uniqueness of the two tasks. By contrast, the comparable typing and pausing patterns indicated a certain degree of consistency in their process of text production. Research on other language combinations also comes to similar conclusions. A recent study by Ma et al. (2022) focused on comparing translation between English and Chinese and English/Chinese paraphrasing. The results again confirmed a more intense cognitive effort in translation, accompanied by a similar pattern of attention shifts in the two tasks. This comparative approach has been widely adopted in relevant studies, mainly process-oriented investigations focusing on decision-making processes and required metacognitive skills, together with several product-oriented explorations studying the use of linking words and translation strategies (Whyatt et al., 2017; Kajzer-Wietrzny, 2019; Whyatt and Naranowicz, 2020). These studies have offered essential insights into the resemblances and divergences between translation and paraphrasing. However, the main limitation of the studies reviewed so far is that a large percentage of them solely probe into the processes of translation and interpreting, failing to taking into account the output products of these two activities (i.e. the final translations and paraphrases) in their analysis and explanation. This limitation calls for an extension of research focus to product quality in order to achieve a complete understanding of the relationship between translation and paraphrasing.

### Methods of product quality assessment in translation and paraphrasing

The evaluation of translation quality has long been a heated topic in Translation studies, probably due to its critical role in the educational setting. Over the years, many methods have emerged and been employed by translation teachers to assess translation quality (Han, 2020). Error analysis has been widely adopted in teaching, a comparably systematic approach to quality assessment, thanks to its specified evaluation criteria on error types (Martínez Melis and Hurtado Albir, 2001). Though this approach outstands in its explicit and detailed diagnosis of the weakness in translations (Eyckmans et al., 2016), some argue that it tends to be simplistic by limiting the focus on linguistic accuracy and leaving out the evaluation of extralinguistic dimensions, such as the pragmatic and communicative functions (Colina, 2009; Martínez Mateo et al., 2017; Han, 2020). To compensate, researchers further implement the rubric scoring method, using rating scales to score different levels of translation performance (Waddington, 2001). This idea of combining accuracy analysis with rubric scoring has been proven feasible (Turner et al., 2010; Lai, 2011), and researchers have constructed several versions of translation assessment rubrics including the assessments of language quality and functional adequacy (Colina, 2008; Angelelli, 2009; Hurtado Albir and Taylor, 2015). However, a critical limitation of these frameworks is that they rely primarily on human judgments, which raises two concerns: subjectivity and practicality. The creation of the rating categories and the descriptive statements for the level of each scale could be subjective, and the evaluation of a given text could also differ considerably between evaluators because of multiple influential factors, for example, personal preference and the order of texts (Colina, 2009; De Sutter et al., 2017). Besides, both error analysis and rubric scoring tend to be time-consuming, especially when facing a large number of translation products (Martínez Melis and Hurtado Albir, 2001). These potential problems highlight the necessity to adopt alternative methods which are more objective and time-efficient. Notably, De Sutter made a crucial attempt by turning to a corpus-based and statistical approach (De Sutter et al., 2017). His study aims to find possible linguistic indicators for the evaluation of translation acceptability by comparing language features in student translations, professional translations, and native writings. Although the results of two case studies reported in this paper fail to reach consistency, this research brings insights into a combined approach to TQA by incorporating objective linguistic indicators into translation teachers’ evaluation rubrics.

Unlike TQA, there seems to be no consensus on the specific criteria for paraphrasing assessment, and the metrics adopted in previous research are mostly borrowed from relevant research areas, for example, machine translation (Chen et al., 2015; Shalamov et al., 2021; Patil et al., 2022). As a result, a unique feature of PQA is the wide use of automatic evaluation models, which measure the paraphrasing quality with the help of NLP (Patil et al., 2022). Nevertheless, these metrics have been criticized for a rather excessive focus on identifying the similarities between the source text and the paraphrased product, probably affected by the highlighted purpose of paraphrases in academic writing to avoid plagiarism (Nicula et al., 2021; Shalamov et al., 2021). Such an approach is not without problems, as the main functions of a paraphrase also include meaning preservation and achievement of communicative purposes (Shalamov et al., 2021). From this perspective, they failed to assess the paraphrasing performance thoroughly. To address this issue, researchers have also made preliminary attempts to propose evaluation rubrics for human assessment based on the criteria from neighboring fields (McCarthy and McNamara, 2008; McCarthy et al., 2009; Chen et al., 2015). For example, Chen et al. (2015) developed a guideline for the holistic evaluation of paraphrasing quality modified from the writing rubrics in TOEFL, with the belief that paraphrasing is considered an essential ability in this task and thus can also be suitable for its evaluation criteria. New evaluation rubrics drawn on metrics from other fields are expected to further enrich the methods of PQA.

As discussed above, current evaluation methods in TQA and PQA have their own strengths while exposing certain weaknesses. TQA has developed a diverse array of methods but seems to place too much reliance on human evaluation, which is inevitably subjective and time-consuming. On the contrary, PQA stands out in its efficient computer evaluation models, but lacks a targeted evaluation for informative and communicative purposes from the human assessment. Notably, the current approaches in TQA and PQA seem to complement each other. This observation enlightens us about a possible way of enriching their evaluation methods by combining automatic evaluation tools with human assessment into a more comprehensive framework. Studies have preliminarily examined whether automatic evaluation models can simulate human assessment on different dimensions. One pioneering study by McCarthy et al. (2009) used the User-Language Paraphrase Corpus to compare human ratings of the paraphrase quality with selected indices in computational assessment. The results highlighted a significant correlation between the two methods in terms of semantic completeness and syntactic similarity, suggesting that computational indices in these dimensions can potentially assist human assessment in paraphrasing quality. Therefore, predictable computational indices in translation quality can be identified and introduced in TQA to assist human assessment. Meanwhile, the scoring rubrics in TQA may also be applicable to paraphrasing evaluation given the shared ground between translation and paraphrasing, especially when the two activities are guided by a similar communicative purpose. More attempts are required for this direction of method improvement in order to achieve a more systematic, comprehensive, and efficient assessment of translation and paraphrasing.

### The current study

In this study, we aim to propose comprehensive frameworks for translation and paraphrasing quality assessment by incorporating NLP indices in textual features with human rating rubrics. With these frameworks, we further investigate the relationship of product quality between translation and paraphrasing tasks. The following two research questions will be answered:

RQ1: Whether and in what way do the textual features extracted by NLP tools correlate with the product quality of translation and paraphrasing?
RQ2: Whether and in what aspects is the product quality in the translation task positively related to that in the paraphrasing task, as indicated by NLP indices and human rating scores?

## Materials and methods

The product data analyzed in this study were collected in a larger project that compared the cognitive processing and translation behaviors between inter-and intra-lingual translation tasks. The present analysis solely focused on the output products from the Chinese-English (C-E) translation task and the English paraphrasing task recorded by Translog II (Carl, 2012).

### Participants

The dataset of translations and paraphrases was collected from 29 participants, containing 24 females and five males aging from 22 to 42 (*M* = 26, *SD* = 4.61). They included 18 first-year MA students majoring in Translation and Interpreting, six PhD students in translation studies with practical experience in translation, and five professional translators with at least 5 years of experience in translation teaching or vocational practice. The apparent disparity in their levels of proficiency assures a considerable variance in translation performance, which helps to demonstrate the tendency of co-variation more clearly if it exists. All the participants were Mandarin native speakers with English as their first foreign language, and they claimed to have a normal or correct-to-normal vision and blind-typing ability. Students and professional translators were paid HKD 200 and HKD 400 for completing the whole experiment, respectively.

### Materials and experiment procedure

Four news reports and four tourism promotional texts of similar length (approximately 160 words) were collected as source texts, half in Chinese and half in English. We made minimized modifications to the texts to ensure reliable comparability in key textual features. The manipulated texts were confirmed equal in the level of readability, textual coherence, and translation difficulty by the ratings of four PhD students in Translation Studies (refer to Ma et al. (2022) for the details of the textual information of the source texts).

**Figure 1:**
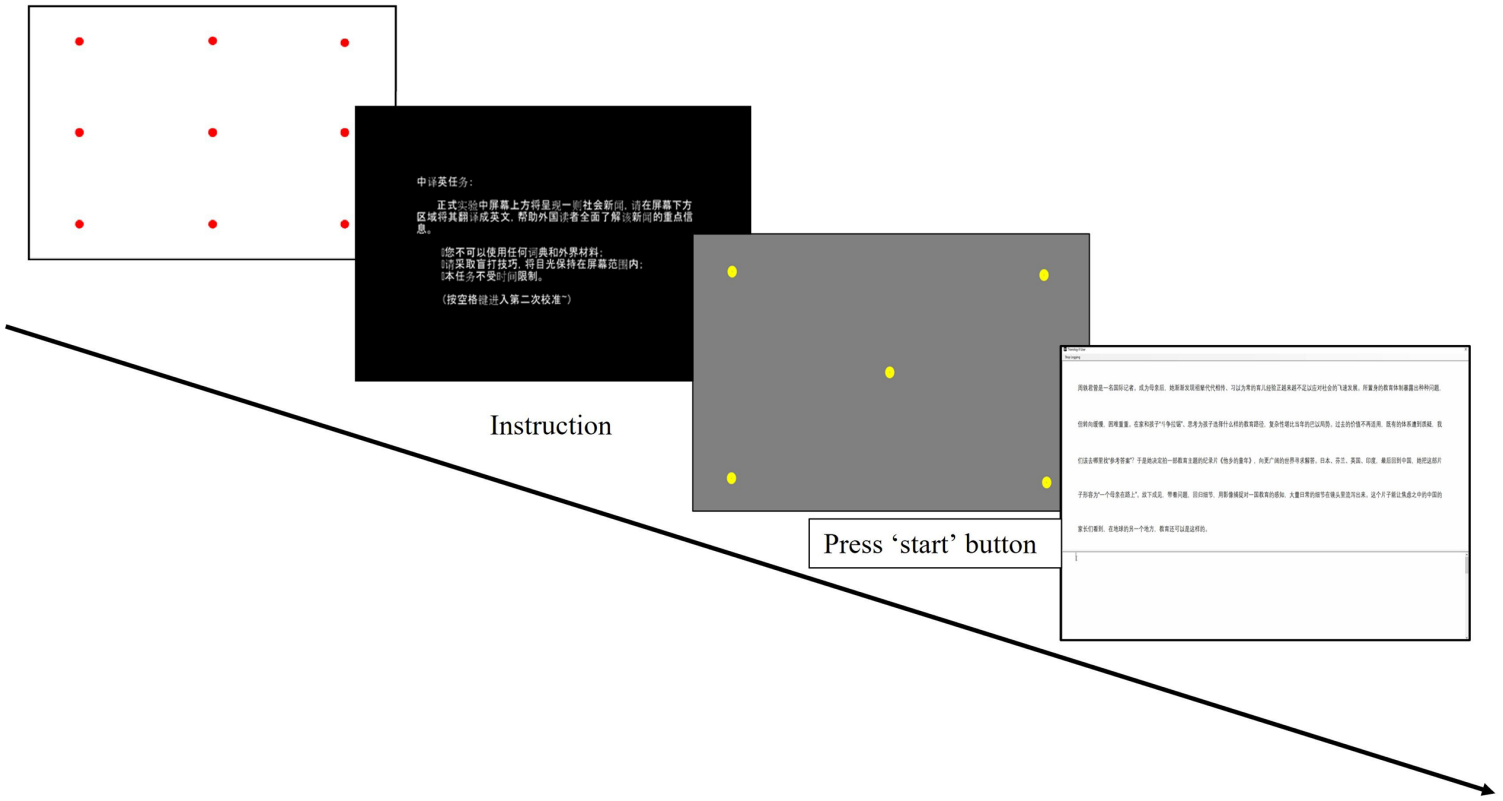
The experimental procedure in one block.

The experiment was conducted in the eye-tracking laboratory at the Department of Chinese and bilingual studies, Hong Kong Polytechnic University. We programmed and performed the translation and paraphrasing tasks on *Tobii Studio 3.4.8* and *Translog II*, with a *Tobii TX300* eye-tracker recording the eye movements during the experiment. The whole experiment contained four tasks, two translation tasks from Chinese to English and vice versa and two Paraphrasing tasks in English and Chinese. The task order was counterbalanced following the Latin Square Design. Participants were assigned two texts of either news reports or tourism promotional texts in each task, and the genre type was also counterbalanced among them. Therefore, each participant produced two C-E translations and two English paraphrases during the experiment. After signing the consent form, they first underwent a practice session to familiarize the techniques and procedure. The experiment was separated into eight blocks, with one text in each block. Figure 1 shows the experimental procedure in one block. The block started with a 9-point eye-tracker calibration. Detailed instruction was provided after that, which specified the anticipated communicative purpose of the target text based on the given genre type. The translation and paraphrasing of a news text aim to pass on the key information to the readers comprehensively. In terms of a tourism text, the aim is to provide a paragraph suitable for the travel brochure to attract potential tourists. Then, they went through another 5-point calibration under stricter validation. By pressing a start button, they began the translation/paraphrasing of the presented text on the computer. During the task, the source text was displayed at the top of the screen, and the target text was produced at the bottom. Participants were asked to minimize their body movement and were allowed to perform the task without time pressure. No external assistance could be used. Instead, they were provided a list of low-frequency words before the experiment to mitigate the comprehension difficulty of unfamiliar words. There was a break of 5 min between two blocks. For each participant, data collection lasted for 2 days, and the completion of the whole experiment required approximately 4 h. A retrospective interview for about 15 min was carried out at the end of each day, which collected participants’ perceptions of task difficulty, self-evaluation of their performance, and explanations of atypical behaviors.

### Data processing

#### Preliminary data cleaning

A total of 116 target texts produced during the C-E translation and E–E paraphrasing tasks were extracted from Translog II, including 58 translations (30 news reports and 28 tourism promotional texts) and 58 paraphrases (28 news reports and 30 tourism promotional texts). We manually checked the original products and revised the typing errors, including misplacement of characters (e.g., “country” mistyped as “conutry”) or mistakes in typing characters because of the similar positions on the keyboard (e.g., “coal” mistyped as “coak”).

#### Product quality evaluation

The quality of each revised text was rated on a 10-point scale by three human raters who were translation teachers with over 5 years of teaching experience in Translation Studies. The 10-point scale represents five product quality levels, from “Poor” to “Excellent,” with two points on each level (see Appendix). This design enables the raters to award a higher score to the relatively better performance within one level (Chen et al., 2015). For the translation quality evaluation, we followed Hu et al. (2021) and adopted the criteria of TEM8 for a holistic assessment and the framework of scoring rubrics in Hurtado Albir (2015) for an analytical assessment. Among the 10 analytical rubrics proposed by Hurtado, we selected eight by categorizing ‘same register’ into the genre appropriateness and removing the ‘cohesion (good use of connectors and referential elements)’ given that our texts might be too short to contain enough indicators. These analytical rubrics reflected three dimensions of quality assessment: meaning expression, composition of target language, and achievement in communicative effects.

For the paraphrasing evaluation, we referred to the metrics in previous studies and formulated our evaluation rubrics following the framework in translation. This paraphrasing assessment also consisted of an overall evaluation of the performance (McCarthy and McNamara, 2008) accompanied by different analytical metrics, including semantic similarity (McCarthy and McNamara, 2008), clarity or readability (Kazemnejad et al., 2020), cohesion (Russo and Salvador, 2004; Russo, 2014), language novelty or diversity (Kazemnejad et al., 2020; Patil et al., 2022), writing quality in orthographical, lexical, and syntactical levels (McCarthy and McNamara, 2008; Kazemnejad et al., 2020), and appropriateness in text genre and task purpose. The assessment of appropriateness evaluates the products in a functional way, which was added considering that our task required the participants to fulfill a communicative goal of exaggerating the effect of specific genres on the readers. Notice that apart from the ‘language novelty’, which is typical only in paraphrasing evaluation, the remaining rubrics in the paraphrasing assessment are almost in line with the evaluation metrics in translation, which enables a comparative analysis of the quality between translation and paraphrasing (Appendix).

Both assessments were conducted using Qualtrics.^1^ After familiarizing the source texts and the scoring rubrics, three raters first took a pretest with eight translations and eight paraphrases. Scores with a difference of three or more points were discussed in order to improve the agreement of evaluation rubrics between the raters (Lu and Wu, 2022). We selected two rating score for further calculation by discarding one that deviates most among the three scores. The interrater reliability of two selected ratings reflected by the Pearson’s correlation showed a moderate consistency (0.51) in translations (Wolf-Quintero et al., 1998) and an existing consistency (0.37) in paraphrases. Then three raters rated the remaining texts independently. Given that the assessment of nearly one hundred texts was time-consuming and effortful, we adopted the design of overlapping rater teams proposed by (Van den Bergh and Eiting, 1989). This method allows raters to rate only part of the texts (e.g., 70 out of 100 texts), and the interrater reliability is estimated by creating overlaps in the sample groups to get at least two scores for each text (Tillema et al., 2012). In line with this design, both translations and paraphrases were divided into three groups, each containing a similar number of texts with a counterbalanced genre type. Each rater was assigned two groups, and we ensured that each text was evaluated by two raters. The two groups assigned to the raters were then combined into one questionnaire, and the presentation order of each translation/paraphrase was randomized. Ratings of the products showed an overall strong consistency, with the Pearson’s correlation reaching 0.62 in translation products and 0.59 in paraphrasing products. The final results of product quality were extracted by averaging two rating scores.

#### Linguistic feature measurement

We further removed the spelling, punctuation, tense, aspect, and number errors in the revised texts before analyzing their linguistic features (Lu, 2012; Hu, 2021). TAALES2.2 and TAASSC1.3.8 (also including L2SCA indices)^2^ were used to extract the lexical and syntactic sophistication indices of texts, respectively (Lu, 2010; Kyle, 2016; Kyle et al., 2018). TAALES offers more than 484 indices of lexical sophistication ranging from lexical frequency, range or contextual diversity, n-gram frequency, academic words, and psycholinguistic properties. These indices are mainly calculated in reference to the Corpus of Contemporary American English (COCA), British National Corpus (BNC), SUBTLEXus corpus, Academic Lists, and MRC psycholinguistic database (Kyle and Crossley, 2015). TAASSC provides a wide range of syntactic indices, including Clause Complexity, Phrase Complexity, Syntactic Sophistication based on the COCA corpus, and L2SCA (14 syntactic complexity indices; see Lu (2010) for details; Kyle, 2016). We selected desired NLP indices in two tools and exported their results for each product text.

#### Correlation analysis

Subsequently, we performed two groups of correlation analyses. The first group focused on the relationship between NLP indicators and the product quality in two tasks. We calculated the correlation coefficients between the result of each NLP indicator and the rating score of each evaluation metric among 58 translations and 58 paraphrases. Subsequently, we narrow our focus to the rating scores, analyzing the correlation in the product quality between translation and paraphrasing. The rating data were first divided into subgroups based on the rating metrics. Then, we looked into each subgroup and calculated the correlation coefficients in the rating score of the corresponding metric between translations and paraphrases among 29 participants. Both analyses adopted the Pearson’s correlation algorithm provided by the SciPy (1.7.3; Virtanen et al., 2020).

## Results

### Correlation between NLP indices and product quality in translation

**Figure 2:**
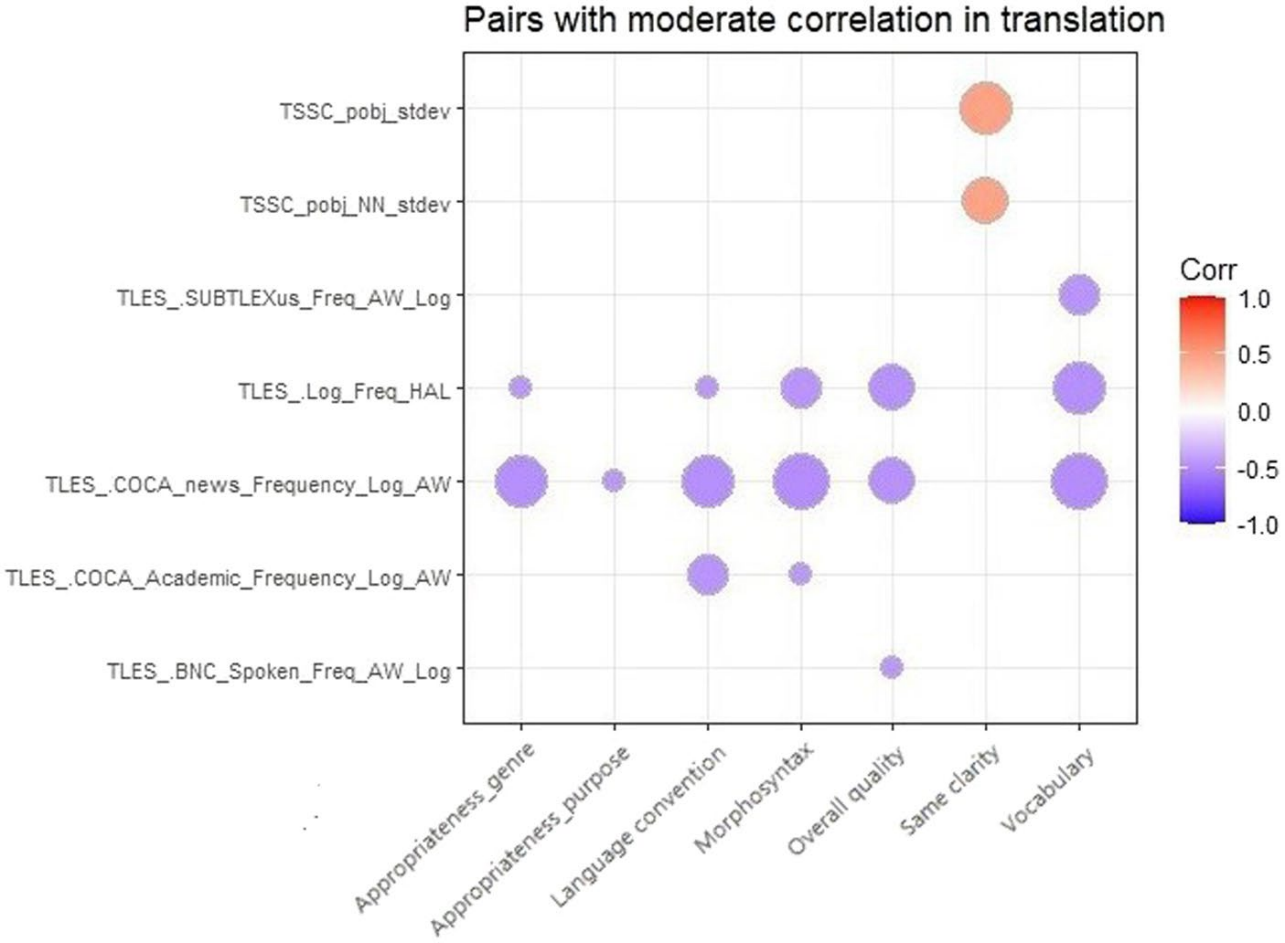
17 pairs of NLP indices and translation quality metrics with a moderate correlation.

Overall, 1,393 pairs of NLP indices and quality metrics were correlated significantly. Figure 2 presents all 17 pairs reaching a moderate correlation. We referred to Wolf-Quintero et al. (1998) and adopted a moderate correlation with.450 < |r| < 0.650. Pairs with a larger circle size in the figure indicated a higher correlation coefficient. Results showed that five indicators of word frequency from TAALES exhibited negative correlations with the overall translation quality and the scores of analytical metrics in two dimensions, the composition of the target language and the achievement of communicative function. Particularly, the COCA_news_Frequency_ Log_AW and Log_Freq_HAL, which measure the mean frequency of words with reference to an English corpus in the news genre and a corpus of conversations to share information online, respectively, showed their close correlations with almost all the scores of analytical metrics. The remaining two indices from TAASSC revealed a positive correlation between the noun phrase (NP) variety of the text and the clarity of the text meaning.

### Correlation between NLP indices and product quality in paraphrasing

**Figure 3:**
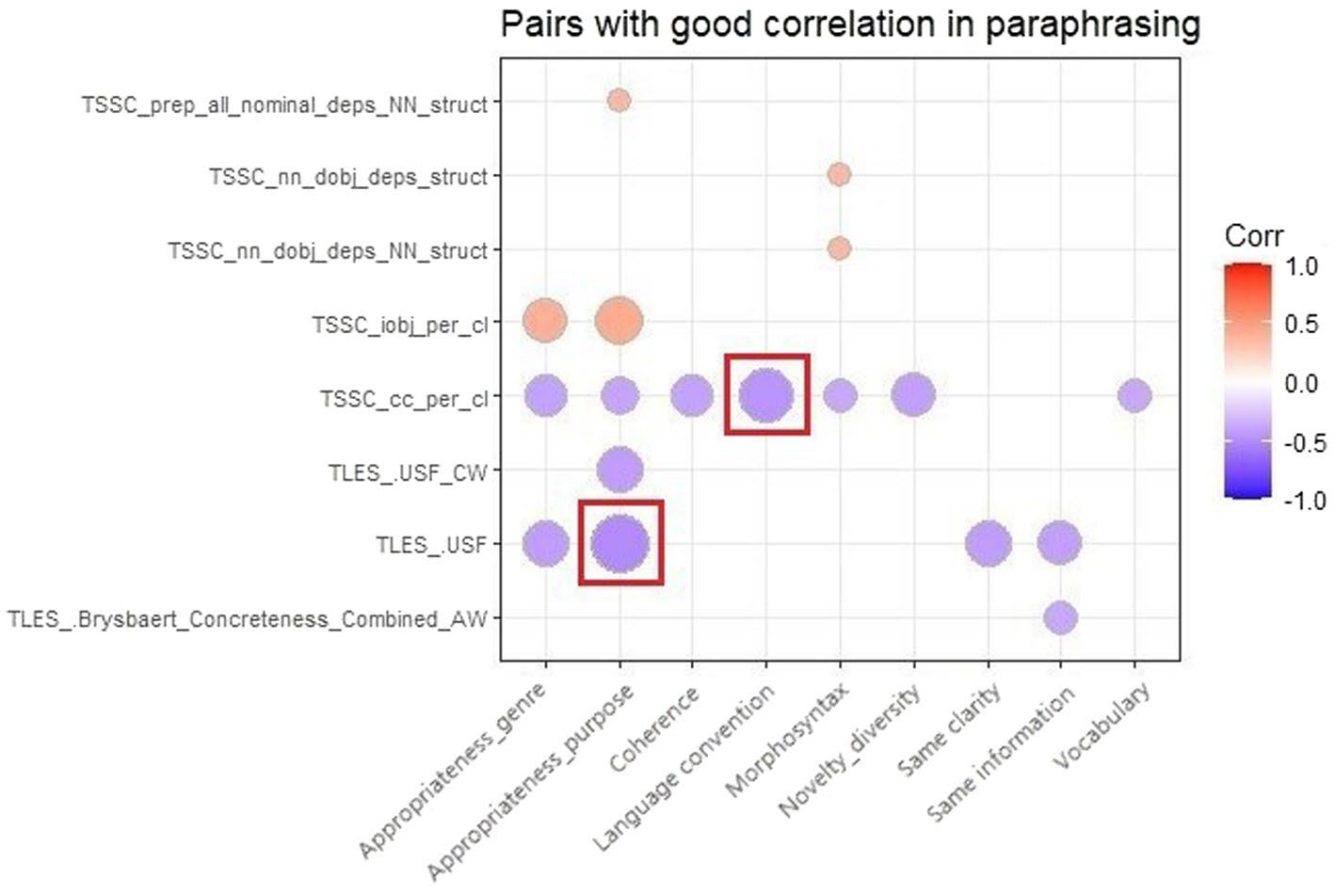
Top 18 pairs of NLP indices and translation quality metrics with strongest correlation.

Generally speaking, the correlation between NLP indices and quality metrics in paraphrasing tended to be weaker than that in translation, as only 279 pairs were significant, and two of them reached a moderate correlation (see the results marked in Figure 3). To achieve a comparative analysis, we extracted the top 18 pairs with the strongest correlation (0.370 < |r| < 0.510), as is shown in Figure 3. Three indices from TAALES were negatively correlated with the paraphrasing quality. Specifically, word concreteness (Brysbaert_Concreteness_Combined_AW) was reported to be closely related to the performance of meaning expression. The contextual distinctiveness of words which measures the semantic diversity of their contexts (USF and USF_CW) revealed significant correlations with both the performance of meaning expression and achievement of communicative functions. As for the indices from TAASSC, a salient correlation was found between clause complexity (indicated by cc_per_cl and iobj_per_cl) and paraphrasing quality in terms of text composition and fulfillment of communicative effects. The remaining three indices representing the NP complexity were also positively correlated with the morphosyntactic-level language quality and the appropriateness of the communicative purpose.

### Shared features in a significant correlation with quality metrics

**Figure 4:**
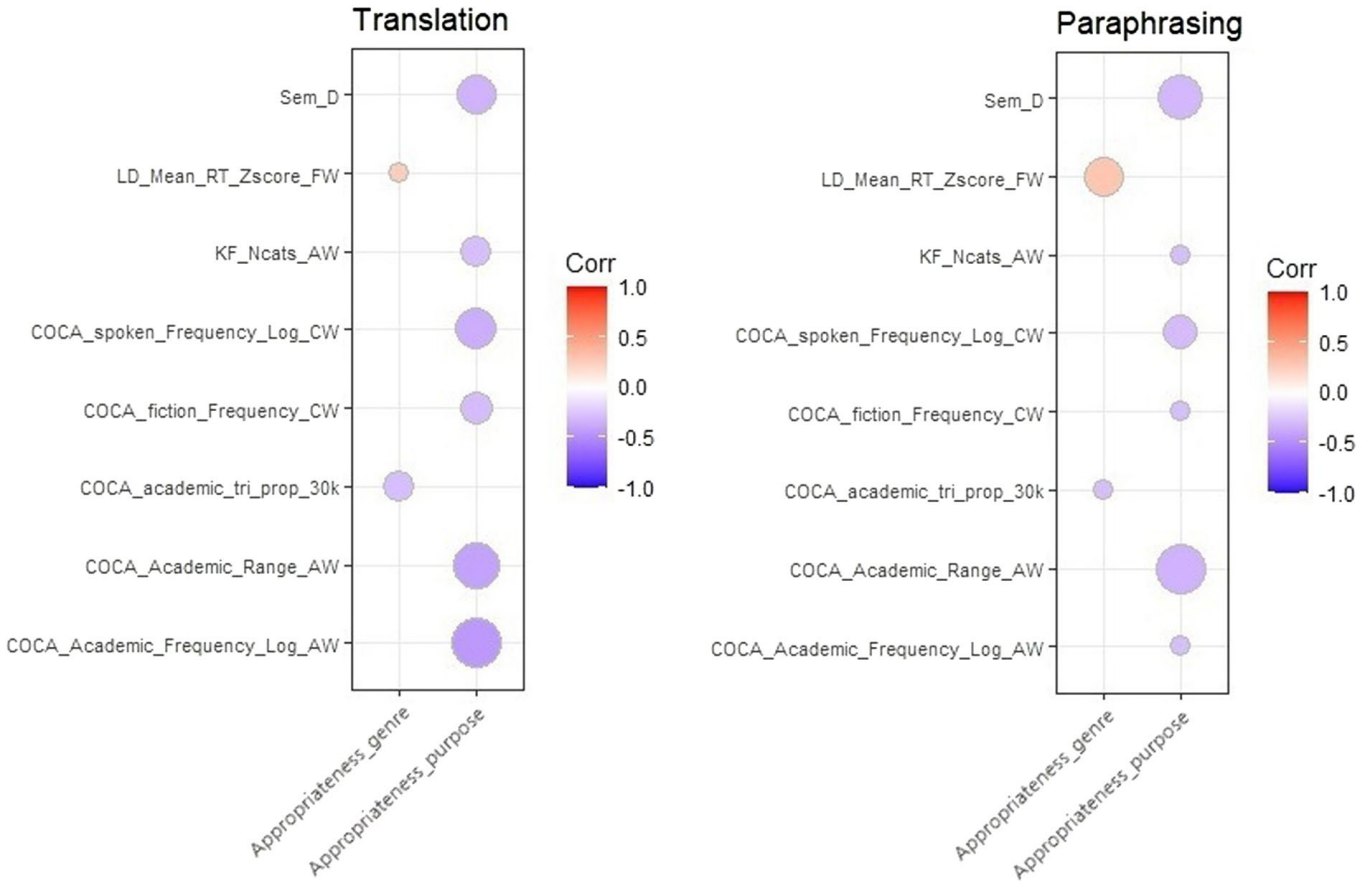
Index-Metric pairs with a significant correlation shared in translation and paraphrasing.

We further looked at the significantly correlated index-metric pairs shared in translation and paraphrasing. As is shown in Figure 4, all the indices were indicators of lexical sophistication from TAALES and were predictable in participants’ performance of communicative function achievement. Products containing more words with a longer reaction time (LD_Mean_RT_Zscore_FW) and more three-word collocations in a low frequency (COCA_academic_tri_prop_30k) led to a significant improvement in genre convention. Better achievements of the communicative effects were predicted by more words with lower frequency (COCA_Academic_Frequency_Log_AW, COCA_fiction_ Frequency_CW, COCA_ spoken_Frequency_Log_CW) and a more restricted range of use in documents (KF_Ncats_AW, COCA_Academic_Range_AW) and contexts (Sem_D).

### Correlation in rating scores between translation and paraphrasing

**Figure 5:**
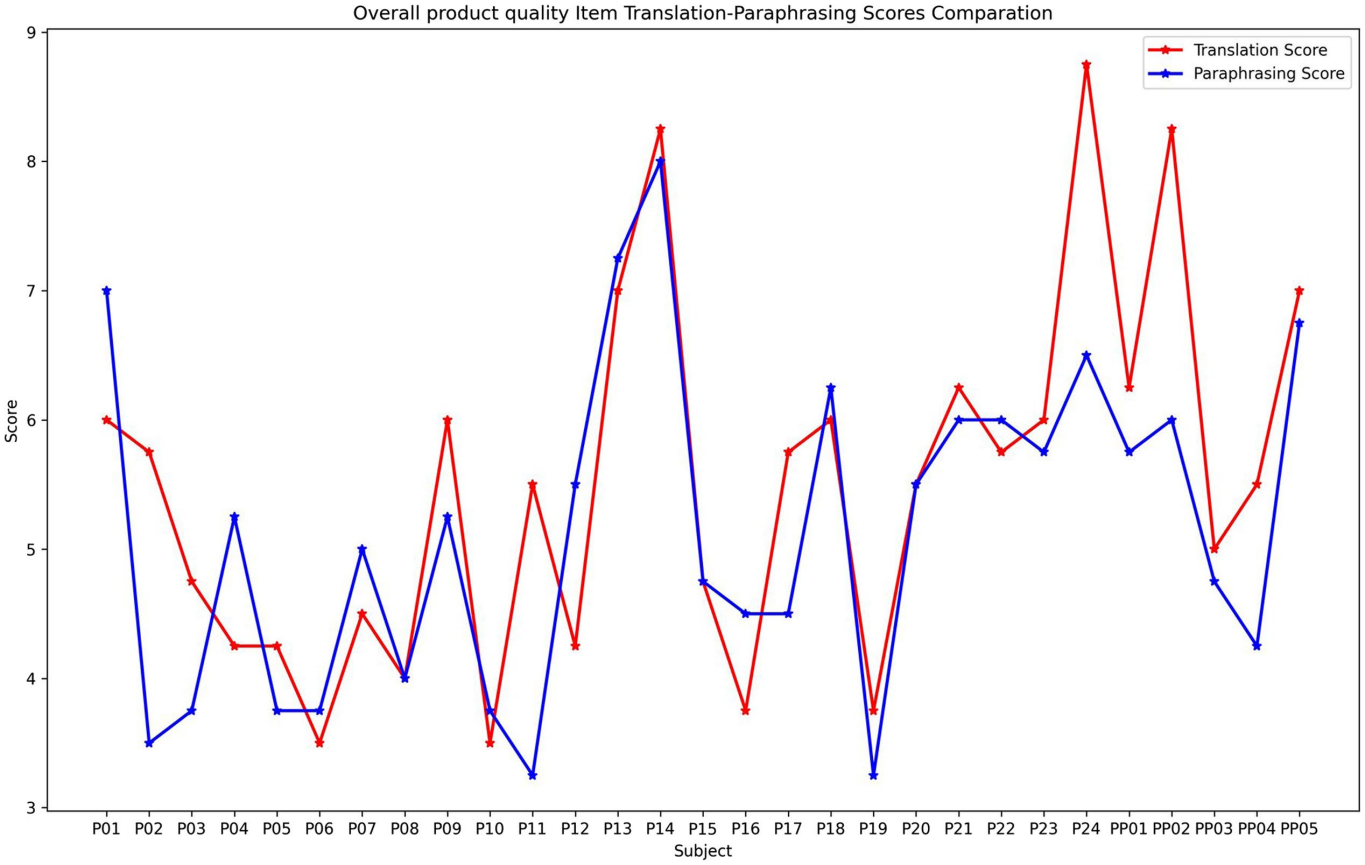
Average rating scores in Overall Product Quality in translation and paraphrasing among 29 participants.

We selected nine metrics of product quality that were matched between the assessments of translations and paraphrases for a comparative analysis (Table 1). Separated analyses of each metric suggested that nearly all the metrics demonstrated a moderate-to-high correlation between translation and paraphrasing, with Pearson’s correlation ranging from 0.622 to 0.777 (Table 2). For example, Figure 5 shows the holistic score of product quality in two tasks among all the participants. Although some participants demonstrated a certain degree of discrepancy in the performance of translation and paraphrasing (e.g., P02, P24, and PP02), the scores in both tasks generally showed an apparent distinction between participants with high proficiency (e.g., P13, P14, and PP05), mediate proficiency (e.g., P18, P22, and P23), and relatively low proficiency (e.g., P06, P10, and P19). Scores of other metrics demonstrated a relatively similar consistency between translation and paraphrasing (see Supplementary material).

**Table 1:** The selected metrics in the comparative analysis of the quality between translation and paraphrasing.

Dimensions	Translation	Paraphrasing
Expression of the meaning	Same information	Same information/semantic similarity
	Same clarity	Same clarity
Composition in the target language	Conventions of written language	Conventions of written language
	Vocabulary	Vocabulary
	Morphosyntax	Morphosyntax
	Coherence	Coherence
Level of communication of the target text	Appropriateness–genre’s conventions	Appropriateness -- genre’s conventions
	Appropriateness–the translation’s purpose and target reader	Appropriateness -- the paraphrase’s purpose and target reader
Overall	Overall translation quality	Overall paraphrasing quality

**Table 2:** Comparative analysis of the correlation in product quality between translation and paraphrasing in each metric.

Metrics	r	p
Same information	0.622	<0.001
Same clarity	0.639	<0.001
Conventions of written language	0.686	<0.001
Vocabulary	0.709	<0.001
Morphosyntax	0.721	<0.001
Coherence	0.749	<0.001
Appropriateness–genre’s conventions	0.777	<0.001
Appropriateness–the communicative purpose and target reader	0.707	<0.001
Overall product quality	0.738	<0.001

## Discussion

Motivated by the gap in the relationship of product quality between translation and paraphrasing and the defects in the current evaluation methods in TQA and PQA, we used NLP tools and human ratings to construct NLP-assisted evaluation frameworks for translation and paraphrasing and compared the product quality between two tasks. Here, we first present the construction of our updated evaluation frameworks and then discuss the similarities and differences in product quality between translation and paraphrasing.

### The development of NLP-assisted evaluation frameworks in TQA and PQA

Our first research question aims to enrich current methods in TQA and PQA, asking whether the indices of textual features from NLP tools can predict product quality successfully. Using TAASSC and TAALES, we found several syntactic and lexical sophistication indices that closely correlate with different dimensions of product quality in translation and paraphrasing, respectively.

In translation, word frequency tends to be a dominant indicator of product quality. Specifically, a translation using more low-frequency words is likely to be well-performed, especially regarding its language quality and the communicative effect. In complement, a good performance in meaning expression can be predicted by a high level of NP variety. In paraphrasing, the concreteness and contextual distinctiveness of words used in products are more closely correlated with the meaning expression, with less concrete and more contextual-specific words signaling a better delivery of the content meaning. Besides, a higher level of the NP and clause complexity tends to suggest a better quality in text composition. The performance of communicative effects in paraphrases can be predicted by both lexical and syntactic-level indicators.

**Figure 6:**
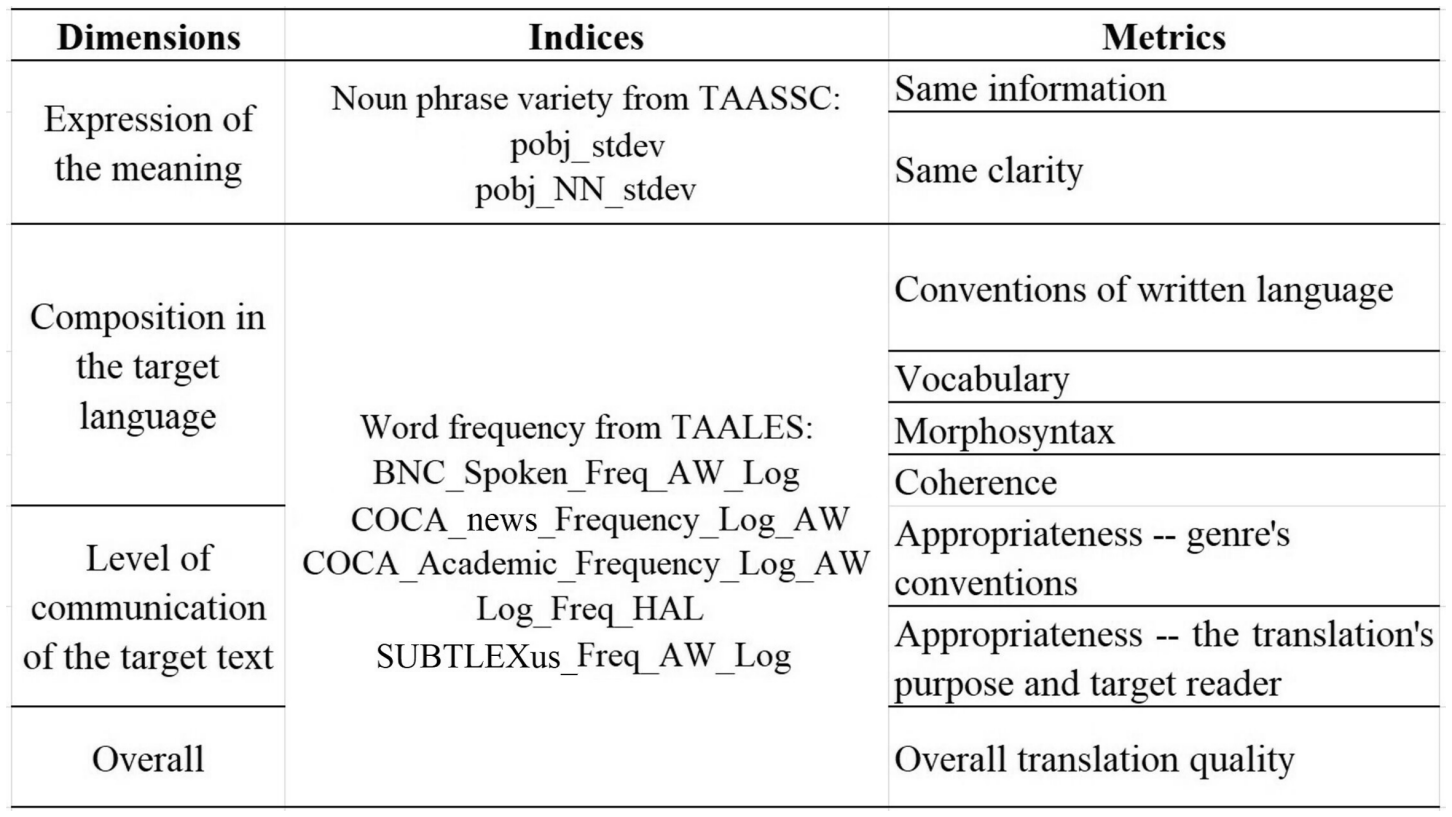
The NLP-assisted Evaluation Framework for TQA.

**Figure 7:**
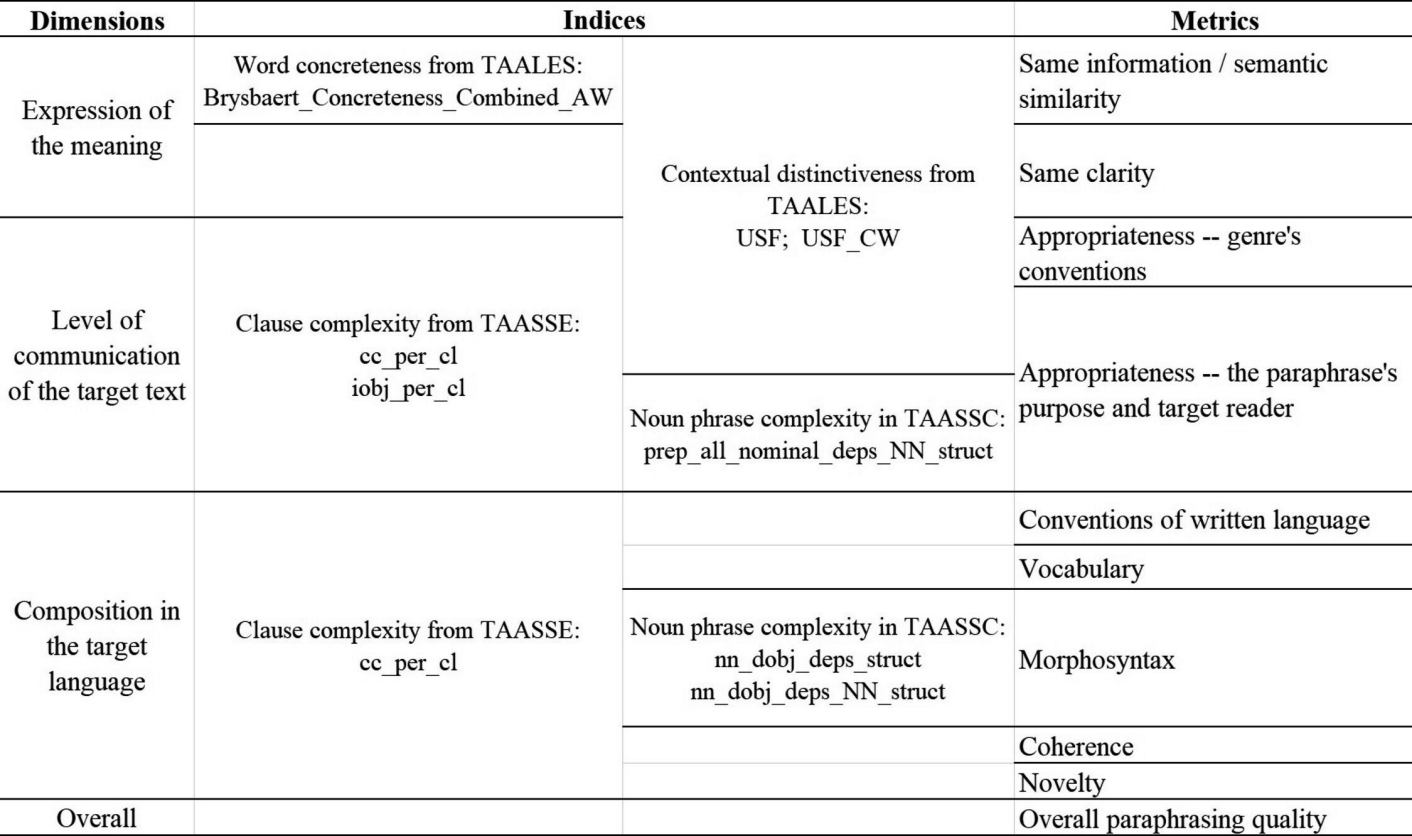
The NLP-assisted Evaluation Framework for PQA.

In summary, all three dimensions in the analytical assessment of product quality, namely meaning expression, language quality, and communicative effects, can find at least one predictive index extracted by NLP tools. Therefore, the original quality evaluation rubrics can be updated by adding these indices accordingly, which may serve as a reference for the teachers to assist in assessing the product quality. Figures 6, 7 represent the new frameworks for quality assessment in translation and paraphrasing, respectively. The proposed evaluation frameworks stand out in their combined measurements with both objective textual features and rubric scoring, which are expected to be effective for the evaluation of translations and paraphrases with a communicative purpose. These evaluation frameworks can be seen as tools for teachers to achieve a more systemic, reliable, and efficient evaluation of translation and paraphrasing quality.

### Comparing product quality between translation and paraphrasing

The proposed evaluation frameworks enable us to compare the product quality between translation and paraphrasing in a more comprehensive way. Results from the textual feature analysis first suggest that the overall predictive effect of textual features tends to be much more robust in translation than paraphrasing. Besides, there exist shared lexical sophistication indices which can distinguish the product quality in both tasks mainly from the perspective of the product’s communicative effects. These indices can measure almost all aspects of lexical sophistication, including word frequency, word range, trigram association strength, and psycholinguistic lexical properties (Kyle and Crossley, 2015). Notably, we further discovered that the predictive weight of the textual features in quality seems to differ between translation and paraphrasing to some extent. Specifically, word frequency is possibly a salient lexical-level measurement to indicate translation performance. In paraphrasing, however, the most predictable indicators tend to measure the words’ psycholinguistic property (concreteness) and their distributional difference (contextual distinctiveness). Moreover, although the performance of two tasks can be predicted by the NP diversity, the quality of paraphrases can also be distinguished by the degree of clause complexity, which may not be applicable in translation.

Regarding the human rating scores, an overall strong positive correlation was observed, with separate analyses confirming relatively strong correlations in both holistic and analytical evaluations. These results imply that, according to the translation teachers, the quality of the translations and paraphrases in the current study is highly related. The participants in our experiment who are proficient in translating tend to also outstand in the performance of the paraphrasing task, and vice versa. Admittedly, the sample size in this correlation analysis is relatively small, which could be a concern for any absolute conclusion. Still, our results could provide preliminary evidence on the consistency of task performance between translation and paraphrasing.

In sum, our findings suggest that there are shared grounds and divergences regarding the product quality between translation and paraphrasing. This conclusion echoes previous studies discussing their similarities and differences from a process-oriented approach (Whyatt et al., 2016; Whyatt and Naranowicz, 2020; Ma et al., 2022). More importantly, the difference in the cognitive load between translation and paraphrasing might be one contributor to the observed divergences in the textual features of their products. Translation has been confirmed to be cognitively more demanding compared to paraphrasing, mainly due to the extra processing cost for bilingual transfer (Whyatt et al., 2016; Ma et al., 2022). Under this circumstance, participants’ language choice is likely to be more affected during translation regardless of their proficiency, resulting in a narrower gap between high-quality and low-quality products. As a result, the vocabulary choices in translations with different qualities can only demonstrate a significant distinction in the breadth of participants’ vocabulary knowledge (e.g., Poor translations are inclined to contain words with a lower frequency). Kyle and Crossley (2015) argued that word frequency measures tend to lack sensitivity to the depth of vocabulary knowledge, such as the psycholinguistic properties or the constraints on use (Schmitt, 2014). For example, words with a similar frequency of occurrence could vary significantly in the values of contextual diversity, which further differentiates their levels of sophistication. It is reasonable that professional translators can perform much better on these lexical features in a less cognitive-demanding condition. Therefore, indicators of these lexical features appear to be more predictive of the paraphrasing quality. Similar to the syntactic level, different levels of translation quality are distinguished mainly by the complexity of NP, whereas paraphrases in different qualities can show diversity in the choice of higher-level linguistic units (e.g., clauses) as well.

Despite the differences mainly in the most predictive textual features, the shared ground in the quality between translation and paraphrasing is still remarkable. The shared lexical indicators, as well as the highly correlated product quality, stand in line with previous findings on the family resemblances in the process between translation and paraphrasing, such as similar mental operations and shared strategies (Zethsen, 2009; Whyatt et al., 2017). Notably, Whyatt (2018) further pointed out that the metacognitive skills used by a translator could even be transferable between procedurally and conceptually similar tasks, and translation and paraphrasing could be one of the examples. These arguments make our findings tenable to some extent, as one with high proficiency in one task is expected to overcome difficulties in another by adopting similar skills and strategies for better performance. Furthermore, these similarities may signal the nature of both translated and paraphrased texts as two typical types of mediated communication in L2 (Baker, 1993). Therefore, another possible explanation of the observed similarities could be that the performance in two tasks reflects people’s general language competence to a great extent. Researchers have claimed that translation and paraphrasing could be crucial signs of language competence in communication (Popescu, 2013; Chen et al., 2015). Indeed, being proficient in these tasks requires a solid foundation of language competence, including linguistic, intercultural, pragmatic, and sociolinguistic competence (Popescu, 2013). This language competence can enable us to perform well in nearly all sorts of language communication tasks varying from translation, paraphrasing, and text summarization to L2 reading and writing. This idea enlightens us about the potential pedagogical value of translation and paraphrasing. For one thing, these translation activities can be applied to second language education as effective exercises to improve students’ reading comprehension ability, strengthen the linguistic and cultural knowledge in L2, and assist in developing L2 writing skills. For another, paraphrasing can also be incorporated directly into the translation training to consolidate students’ language and translation competence.

## Conclusion

The current study made a preliminary attempt to improve the evaluation methods in TQA and PQA and further enrich our knowledge of the similarities and differences in product quality between translation and paraphrasing. We constructed the integrated evaluation frameworks for translations and paraphrases by incorporating objective indices of textual features from NLP tools into human evaluation rubrics. The NLP-assisted evaluation frameworks for translation and paraphrasing are expected to assist translation teachers in a more systemic, reliable, and efficient evaluation of translation and paraphrasing quality. Following these frameworks, we further compared the product quality between translation and paraphrasing. A close resemblance was observed in the performances of the two tasks, as their products shared a set of quality-related features in lexical sophistication and show strong correlations in quality according to all the rating metrics. This consistency between translation and paraphrasing performance seems to indicate a vital role of shared language competence and metacognitive skills in different translation activities. Meanwhile, certain lexical and syntactic indices tend to show a different predictive power on translation and paraphrasing quality. Indices representing word frequency and NP variety were highly correlated with the translation quality. In contrast, indicators in lexical concreteness, word contextual distinctiveness, and the complexity in clause and NP were more predictable in paraphrasing quality. These divergences can be attributed to the different processing costs required by the two tasks. Our findings can deepen our understanding of the similarities and differences between translation and paraphrasing and shed light on the pedagogical application of translation activities in classroom settings to enhance language competence.

This study also has several limitations. One is the small sample size since the product data came from an empirical experiment with limited participants. Besides, the source text genre included in our study is also limited to News and Tourism. This may partially account for the observed remarkable correlation between the translation quality and the word frequency indices from a newspaper corpus. Therefore, studies in the future can adopt a corpus-based approach by including more texts and more types of genres to verify our findings. Last but not least, our preliminary construction of the NLP-assisted rating frameworks for quality assessment needs further verification and development. A final goal could be to identify predictable indices through a large amount of data and establish an online automatic assessment website to evaluate translation and paraphrasing quality.

## Data availability statement

The datasets presented in this study can be found in online repositories. The names of the repository/repositories and accession number(s) can be found at: https://osf.io/yh7e5/

## Ethics statement

The studies involving human participants were reviewed and approved by The Polytechnic Institutional Review Board (IRB; Ref No. HSEARS20161229004). The participants provided their written informed consent to participate in this study.

## Author contributions

TH conducted the experiment and wrote the first draft of the manuscript. DL contributed to the data collection and the revision of manuscript. XM helped with preparing the experimental materials and revising the manuscript. NH contributed to the statistical analysis and visualization. All authors contributed to the article and approved the submitted version.

## Funding

This study is supported by a General Research Fund (GRF) grant (Ref No. 15600419) from the Research Grants Council of Hong Kong for DL, and partially supported by the Social Science Funding of Jiangsu Province (No. 21YYC006), Jiangsu Shuangchuang Talent Program (No. JSSCBS20210101), and the Fundamental Research Funds for the Central Universities (No. 2242021R40017) for XM.

## Conflict of interest

The authors declare that the research was conducted in the absence of any commercial or financial relationships that could be construed as a potential conflict of interest.

## Publisher’s note

All claims expressed in this article are solely those of the authors and do not necessarily represent those of their affiliated organizations, or those of the publisher, the editors and the reviewers. Any product that may be evaluated in this article, or claim that may be made by its manufacturer, is not guaranteed or endorsed by the publisher.

## Footnotes

^1^Qualtrics website: https://www.qualtrics.com/au/.

^2^For details of these two tools, refer to the website: https://www.linguisticanalysistools.org/
