# Supplementary material for: Comparing product quality between translation and paraphrasing: Using NLP-assisted evaluation frameworks
Source: Front Psychol. 2022 Nov 25;13:1048132. doi: 10.3389/fpsyg.2022.1048132 (PMC9732433; doi:10.3389/fpsyg.2022.1048132)
Supplement: Supplementary file 1 [file Data_Sheet_1.docx]

**Appendix 1 Quality Assessment Rubrics for translation and paraphrasing**

**Translation assessment rubric:**

10-point rating scale:

9-10 Excellent

7-8 Good

5-6 Passable

3-4 Inadequate

1-2 Poor

9 Metrics:

**Expression of the meaning of the original text**:

- **Same information**: the meaning is accurate and loyal
- **Same clarity**: the meaning is understandable and clear

**Composition in the target language**:

- **Conventions of the written language**: correct orthography and grammar
- **Vocabulary**: appropriateness and richness
- **Morphosyntax**: good use of syntactic structures, prepositions, verb tenses and modes, etc.
- **Coherence**: ideas well organized and clearly presented; good use of connectors.

**Level of communication of the target text**:

- **Appropriateness in terms of the genre’s conventions**: the text style is appropriate
- **Appropriateness in terms of the translation’s purpose and target reader**

The translation purpose in news text: pass on the key information to the foreign readers comprehensively;

The translation purpose in tourism text: provide a paragraph suitable for the travel brochure to attract potential tourists

- **Overall evaluation**:


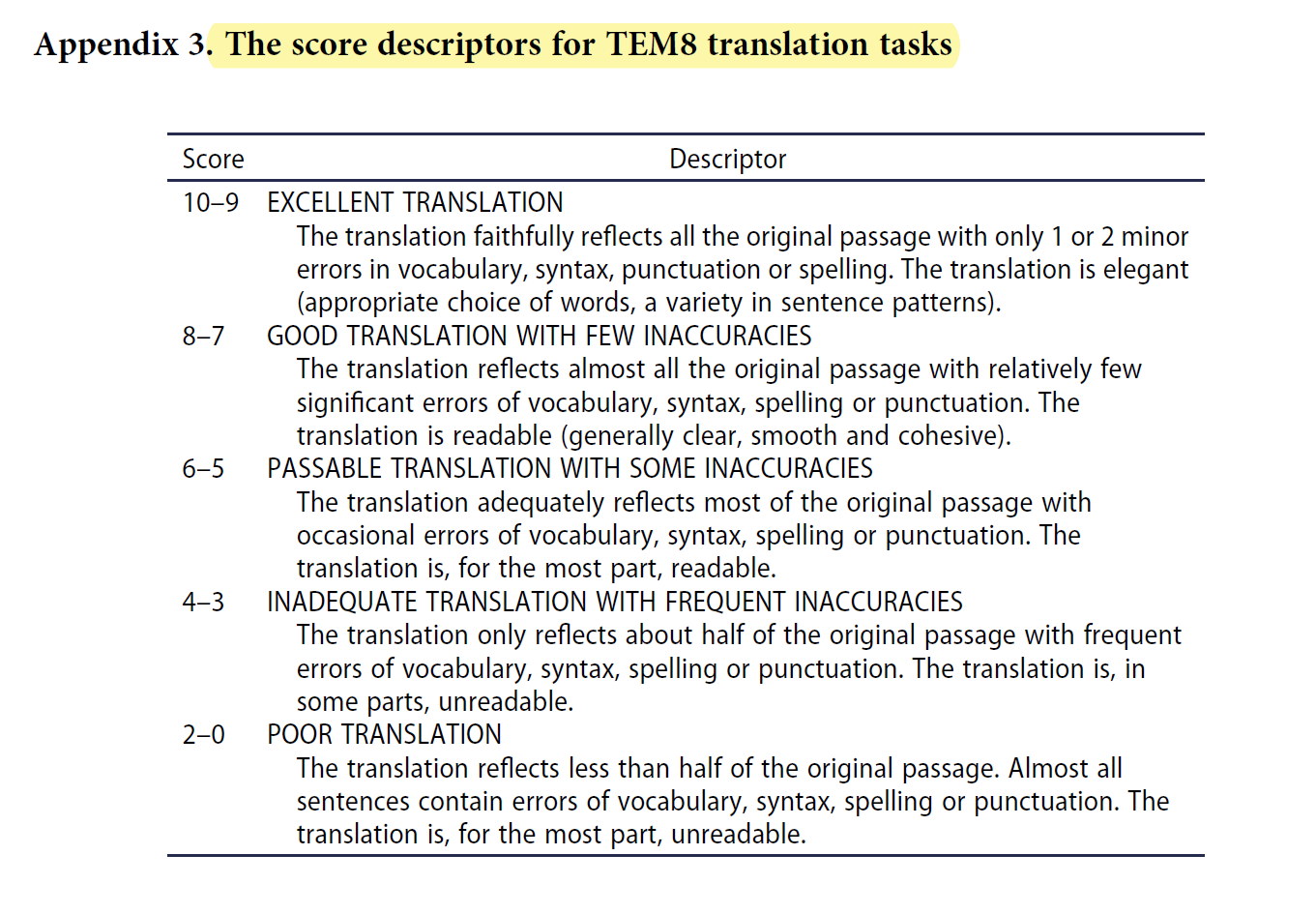


**Paraphrase assessment rubric:**

10-point rating scale:

9-10 Excellent

7-8 Good

5-6 Passable

3-4 Inadequate

1-2 Poor

10 Metrics:

**Expression of the meaning of the original text**:

- **Same information or semantic similarity**: consistency in meaning
- **Same clarity:** the meaning is understandable and clear

**Composition in the target language**:

- **Conventions of the written language**: correct orthography and grammar
- **Vocabulary**: appropriateness and richness
- **Morphosyntax**: good use of syntactic structures, prepositions, verb tenses and modes, etc.
- **Coherence**: ideas well organized and clearly presented
- **Novelty or diversity**: using different words, phrases, or sentences but without changing the meaning considerably

**Level of communication of the target text**:

- **Appropriateness in terms of the genre’s conventions:** the text style is appropriate, and the paraphrase does not change the genre type of the source text.
- **Appropriateness in terms of the paraphrase’s purpose and target reader**

The paraphrase purpose in news text: pass on the key information to the foreign readers comprehensively;

The paraphrase purpose in tourism text: provide a paragraph suitable for the travel brochure to attract potential tourists

- **Overall evaluation**:

An over-arching evaluation of the product, taking into account semantic-overlap, lexical and syntactical variations, writing quality, the fulfillment of the task purpose, and the appropriateness of target readers.
