# Supplementary material for: Comparing product quality between translation and paraphrasing: Using NLP-assisted evaluation frameworks
Source: Front Psychol. 2022 Nov 25;13:1048132. doi: 10.3389/fpsyg.2022.1048132 (PMC9732433; doi:10.3389/fpsyg.2022.1048132)
Supplement: Supplementary file 2 [file Data_Sheet_2.docx]

Scores of eight analytical metrics in translation and paraphrasing among 29 participants


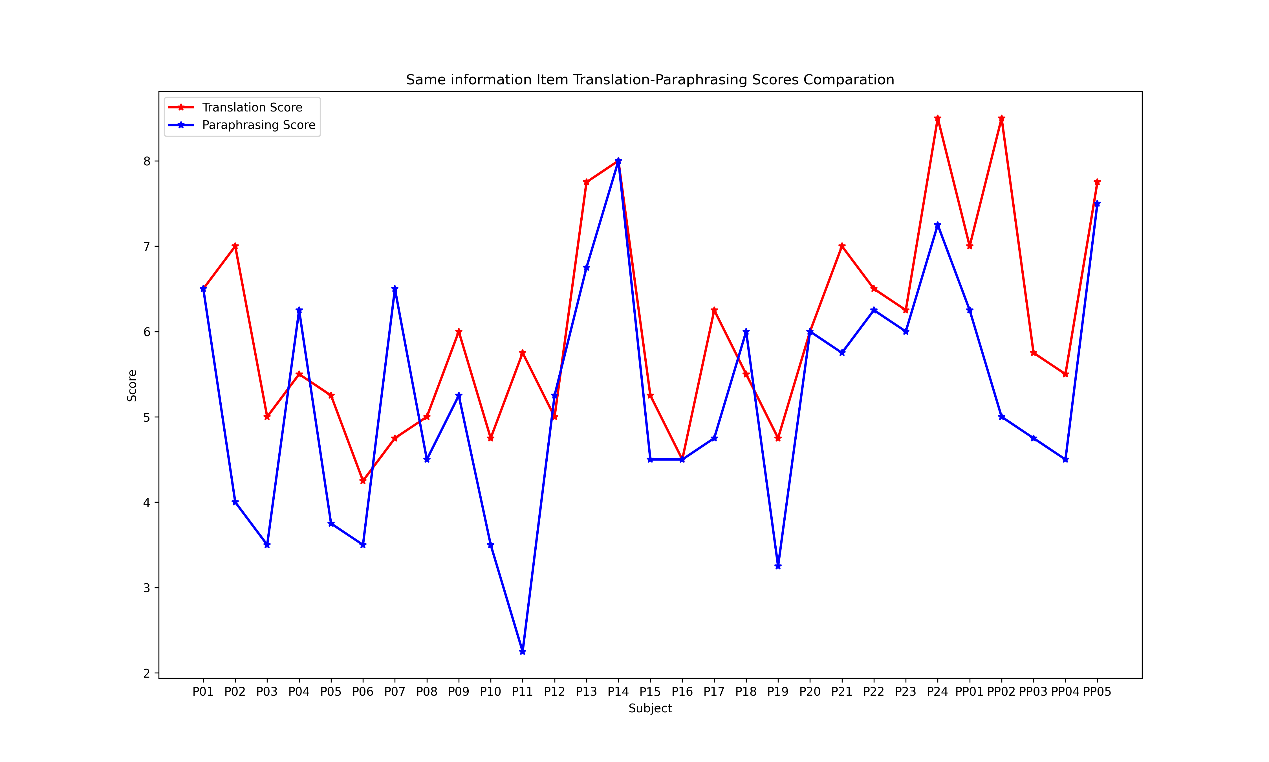


Rating scores of Same information between translation and paraphrasing


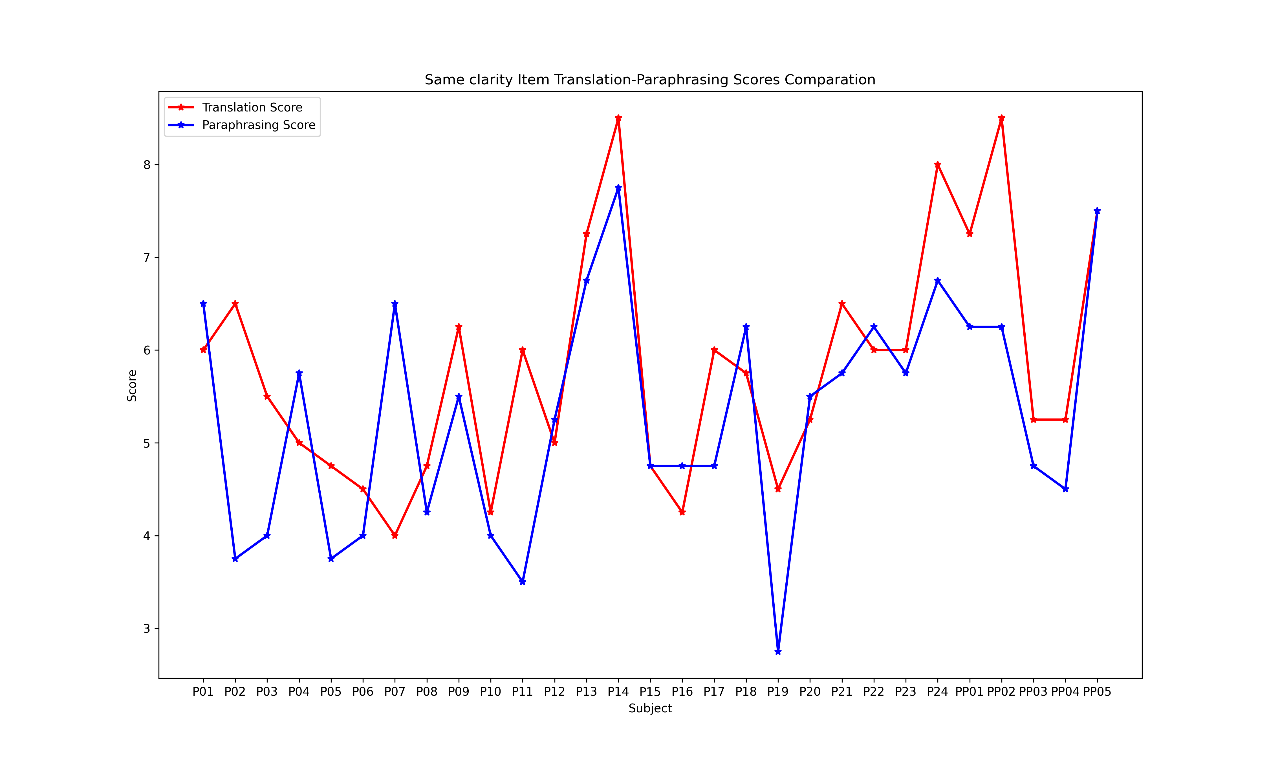


Rating scores of Same clarity between translation and paraphrasing


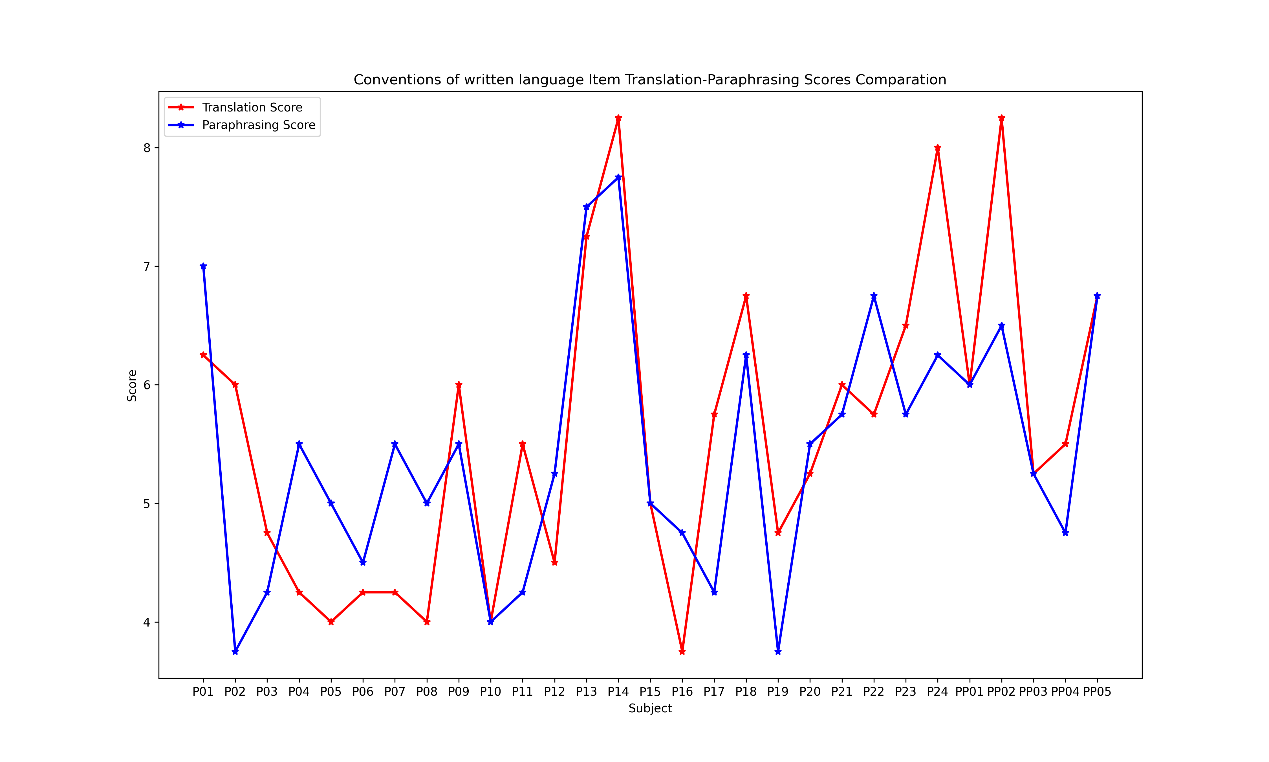


Rating scores of Conventions of written language between translation and paraphrasing


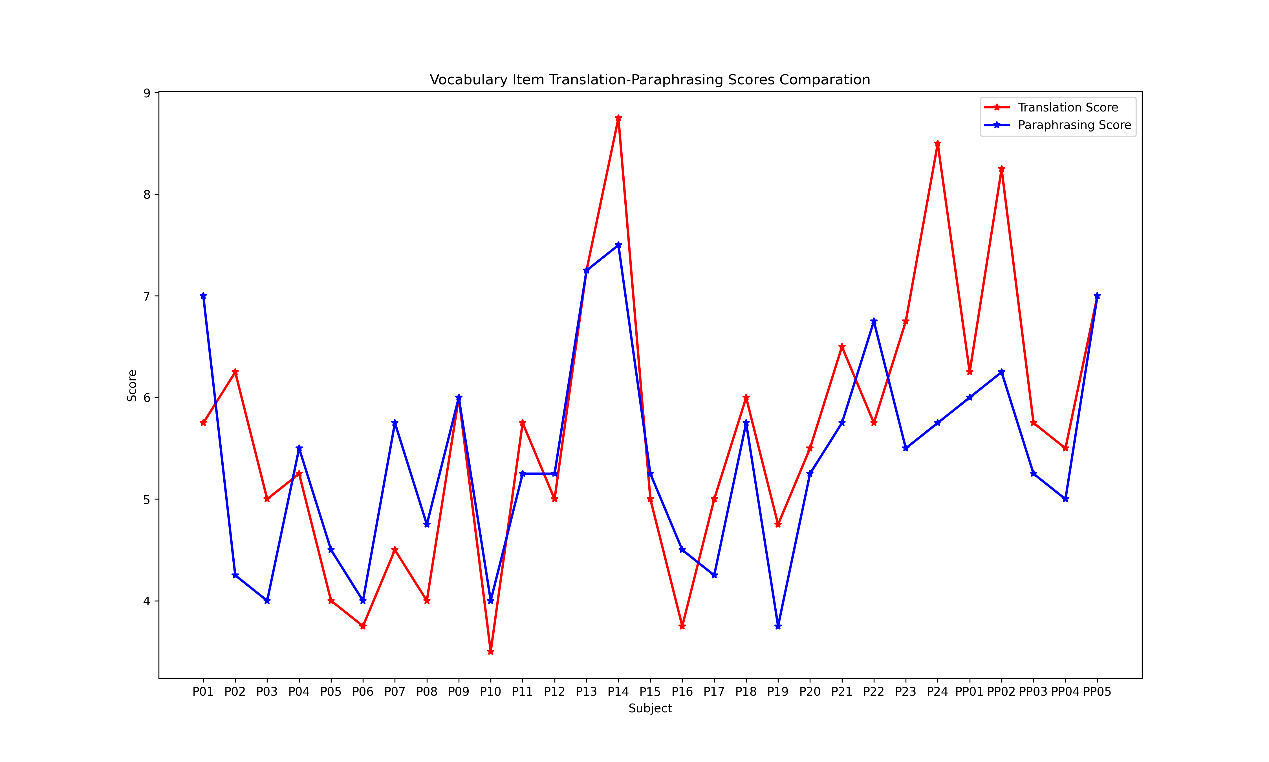


Rating scores of Vocabulary between translation and paraphrasing


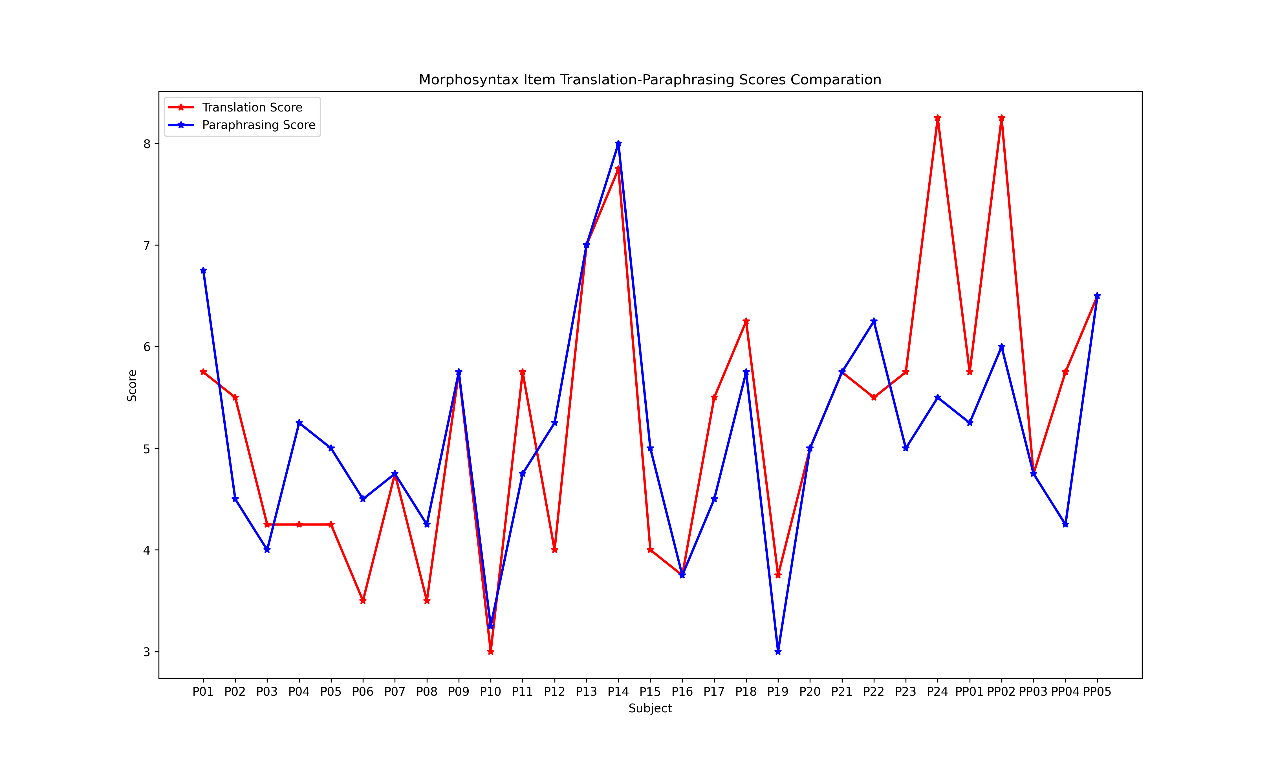


Rating scores of Morphosyntax between translation and paraphrasing


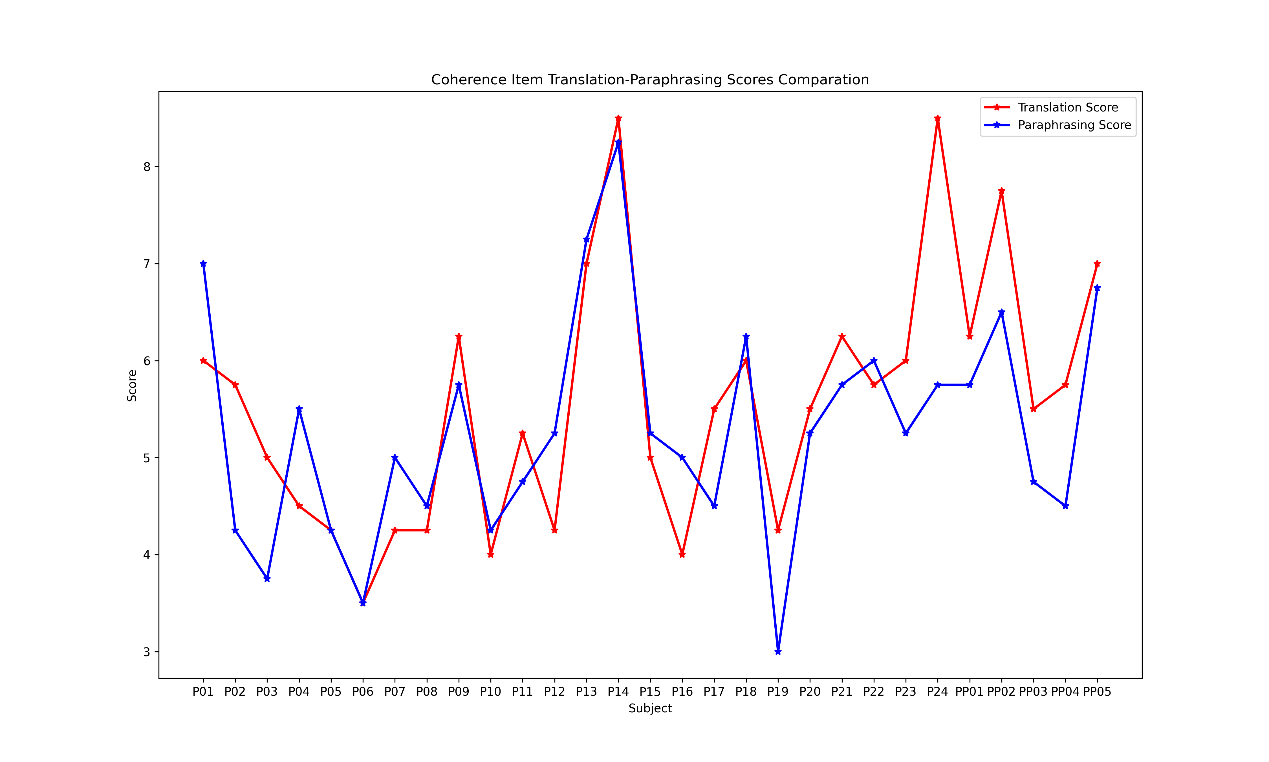


Rating scores of Coherence between translation and paraphrasing


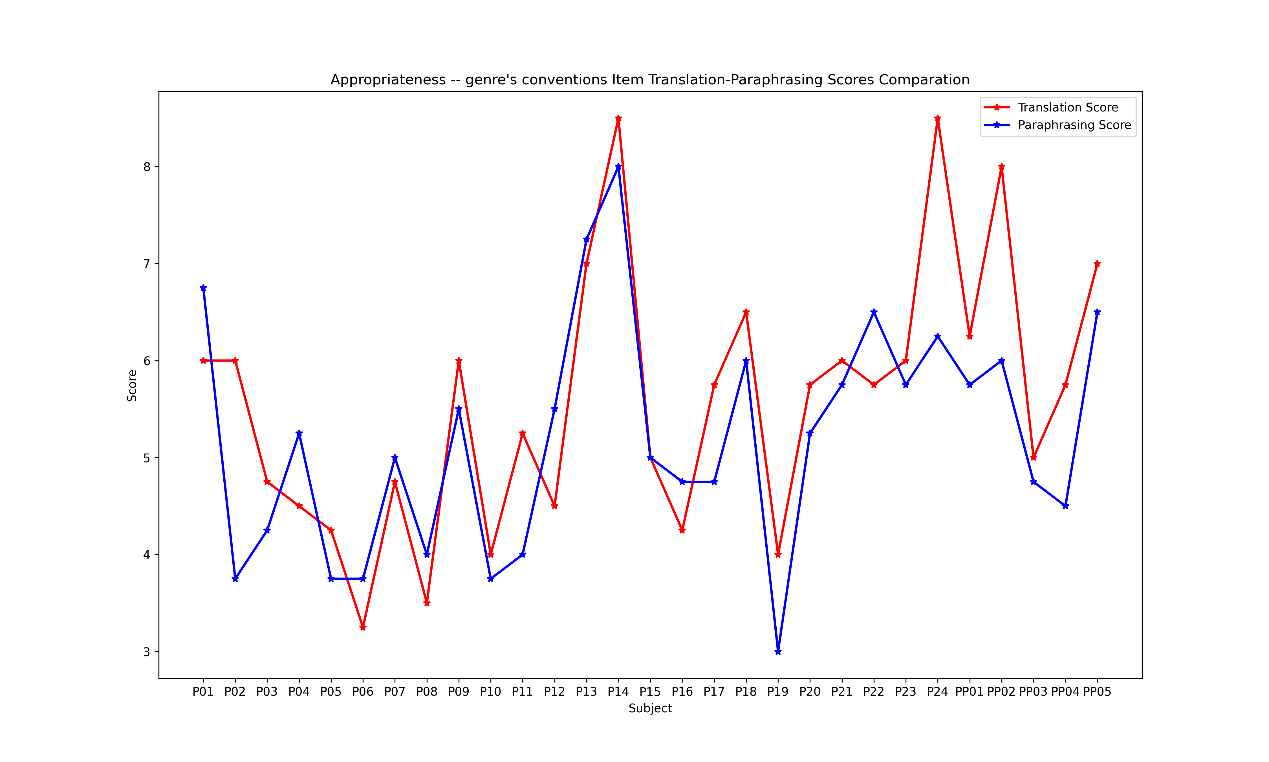


Rating scores of Appropriateness -- genre's conventions between translation and paraphrasing


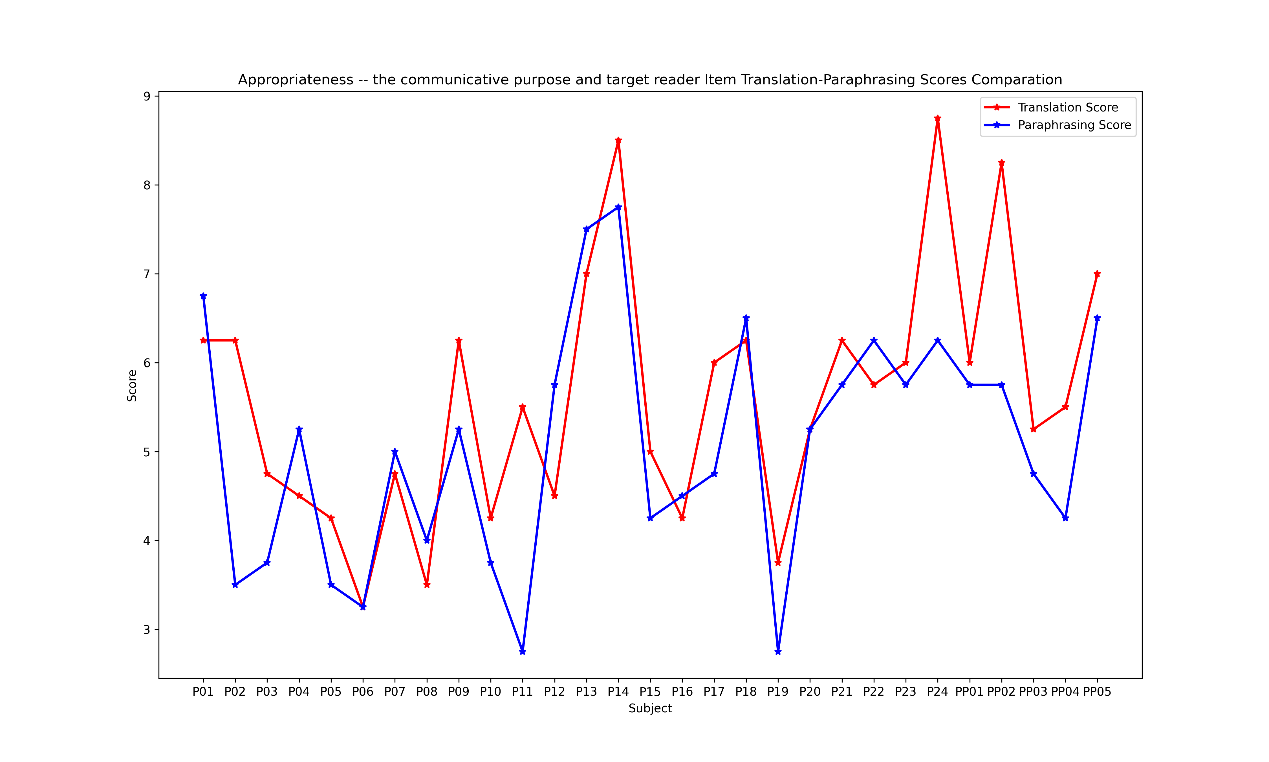


Rating scores of Appropriateness -- the communicative purpose and target reader between translation and paraphrasing
